# Natural Protein Kinase Inhibitors, Staurosporine, and Chelerythrine Suppress Wheat Blast Disease Caused by *Magnaporthe oryzae Triticum*

**DOI:** 10.3390/microorganisms10061186

**Published:** 2022-06-09

**Authors:** Moutoshi Chakraborty, S. M. Fajle Rabby, Dipali Rani Gupta, Mahfuzur Rahman, Sanjoy Kumar Paul, Nur Uddin Mahmud, Abdullah Al Mahbub Rahat, Ljupcho Jankuloski, Tofazzal Islam

**Affiliations:** 1 Institute of Biotechnology and Genetic Engineering (IBGE), Bangabandhu Sheikh Mujibur Rahman Agricultural University, Gazipur 1706, Bangladesh; moutoshi1313@gmail.com (M.C.); fajle.bge@gmail.com (S.M.F.R.); drgupta80@gmail.com (D.R.G.); skpaul_bt@yahoo.com (S.K.P.); numahmud_btl@yahoo.com (N.U.M.); rahatsau@gmail.com (A.A.M.R.); 2 Extension Service, Davis College of Agriculture, West Virginia University, Morgantown, WV 26506, USA; mm.rahman@mail.wvu.edu; 3 Plant Breeding and Genetics Section, Joint FAO/IAEA Centre, International Atomic Energy Agency, 1400 Vienna, Austria; l.jankuloski@iaea.org

**Keywords:** antifungal secondary metabolites, alkaloids, biocontrol, abnormal germ tube, abnormal appressoria, wheat blast

## Abstract

Protein kinases (PKs), being key regulatory enzymes of a wide range of signaling pathways, are potential targets for antifungal agents. Wheat blast disease, caused by *Magnaporthe oryzae Triticum* (MoT), is an existential threat to world food security. During the screening process of natural metabolites against MoT fungus, we find that two protein kinase inhibitors, staurosporine and chelerythrine chloride, remarkably inhibit MoT hyphal growth. This study further investigates the effects of staurosporine and chelerythrine chloride on MoT hyphal growth, conidia production, and development as well as wheat blast inhibition in comparison to a commercial fungicide, Nativo^®^75WG. The growth of MoT mycelia is significantly inhibited by these compounds in a dose-dependent manner. These natural compounds greatly reduce conidia production in MoT mycelia along with suppression of conidial germination and triggered lysis, resulting in deformed germ tubes and appressoria. These metabolites greatly suppress blast development in artificially inoculated wheat plants in the field. This is the first report of the antagonistic effect of these two natural PKC inhibitory alkaloids on MoT fungal developmental processes in vitro and suppression of wheat blast disease on both leaves and spikes in vivo. Further research is needed to identify their precise mechanism of action to consider them as biopesticides or lead compounds for controlling wheat blast.

## 1. Introduction

Protein kinase C (PKC) is a serine/threonine kinase that is found in all eukaryotes and plays a key role in a wide range of signaling pathways, including cell growth and proliferation, sensitivity to external stimuli, DNA damage response, metabolic regulation, and death [1,2]. The PKC is a promising target for antifungal agents due to its functional significance [3]. A large number of natural protein kinase inhibitors have a relatively higher level of selectivity for a specific protein kinase [4]. Almost all of the protein kinase inhibitors are ATP-competitive, but their specificity is based on the interactions with residues that directly bind to the ATP [5,6]. During the screening of several natural secondary metabolites against the deadliest phytopathogen wheat blast fungus *Magnaporthe oryzae Triticum* (MoT) pathotype, we find potential antifungal activity from two PKC inhibitors, staurosporine and chelerythrine chloride.

Staurosporine is an indolo [2,3-a] carbazole alkaloid compound originally extracted from *Streptomyces staurosporeus* and *Streptomyces roseoflavus* strains [7,8,9]. It is known as a protein kinase inhibitor that regulates ATP binding to kinases and impacts cell viability through engaging in the apoptotic process [10,11,12]. Chelerythrine chloride, a benzophenanthridine alkaloid, is another highly specific PKC inhibitor. It was extracted from herbal plants, including *Chelidonium majus* and *Macleaya cordata* [13,14,15]. Chelerythrine appears to be a powerful and selective inhibitor of group A and group B kinases by competing with the conserved catalytic domains of PKC [16,17,18]. Staurosporine and chelerythrine chloride have biological significance because the alkaloids are well known for their antifungal, antiviral, anticancer, anti-inflammation, and anti-tumor effects [19,20,21,22]. Staurosporine and chelerythrine have also been shown to have antifungal properties against *Trichoderma viride*, *Fusarium oxysporum*, *Vermicularia capsica*, and *Verticillium dahlia* [23,24]. Staurosporine and chelerythrine chloride were found recently in our lab study to inhibit MoT.

The MoT is a hemi-biotrophic ascomycetous fungal pathogen of wheat, which causes the most severe wheat blast disease [25,26]. In 1985, the disease was initially detected in the Brazilian state of Paraná [25], and it quickly spread to adjacent countries like Bolivia [27], Paraguay [28], and Argentina [29,30]. Then, wheat blast has posed a severe threat to the wheat production in the southern cone of South America’s tropics and subtropics. Wheat blast made its first appearance outside of South America in Bangladesh in 2016 [26,31], and it has the possibility to spread to other main wheat-growing neighboring countries like Pakistan, India, and China, where wheat is a principal food crop for a billion people [32]. It has also recently been detected in Zambia in Africa [33].

The infection cycle of MoT begins when three-celled hyaline conidium lands on wheat leaf and adheres to it via an adhesive. Then, it germinates, forming a slender germ tube with an appressorium on the tip. At the bottom of the appressorium, a thin penetration peg forms, collapsing the cuticle and permitting entrance into the wheat epidermis. Bulbous, vigorous mycelium permeates wheat plasma membranes and invades epidermal cells, causing plant tissue invasion [34,35,36]. During all phases of growth, it affects the wheat plant’s aerial portions, particularly leaves, spikes, stems, and nodes [37,38,39]. MoT predominantly infects spikes. Bleaching of infected spikes results in a malformed seed or no seed production. The severely affected wheat head may die, leading to a significant drop in production. The most prevalent symptom is the bleaching of spikelets and the whole head [26,40,41]. Conidia of this fungal pathogen are not known to disperse far by wind [42], but seedborne inoculum probably have aided the pathogen’s long-distance dissemination and allowed it to infiltrate other agroecosystems in South America, Southeast Asia, and now Africa [26,30,33,43,44,45]. Contaminated agricultural wastes and seeds may also harbour this fungus [46].

Because of the development of synthetic pesticide-resistant fungi, detrimental impacts of residual chemicals on ecosystem, excessive rate of application with several outdated compounds, and rising regulatory needs, there is a constant need for novel crop protection agents against emerging pathogens [47,48,49]. The purpose of the natural product search is to find new, bioactive metabolites that could be used as novel commercial products or as new lead compounds for chemical synthesis.

Microorganisms and plant-derived secondary metabolites have received increasing interest in research as tools for pest and disease control in agriculture [36,50]. They have complex structures with a specific mechanism of action, which may be amenable to a higher degree of application selectivity. In the biosphere, a microbial or plant-based pesticide degrades quickly, resulting in low residue levels, and it offers several advantages as pesticides or fungicides [51,52]. We have found two natural alkaloids, staurosporine and chelerythrine chloride, with an inhibitory effect against wheat blast fungus. These compounds may be a better alternative for controlling wheat blast disease. To date, no data has been published on the antimicrobial properties of alkaloid antibiotics for managing wheat blast disease. This is, to the best of our knowledge, the first reporting of alkaloid antibiotics being used to control the damaging wheat blast disease with biorational compounds. The specific objectives of this study were to (i) assess the effects of staurosporine and chelerythrine chloride on MoT mycelial growth; (ii) evaluate their inhibitory effects on conidia production, germination, and developmental transitions; (iii) evaluate the efficacy of these alkaloids tocontrol wheat blast disease in vivo; and (iv) compare the efficiency of these two alkaloids with a commercial fungicide, Nativo^®^75WG.

## 2. Materials and Methods

### 2.1. Fungal Strain, Growth Medium, and Plant Materials

In 2016, the MoT strain BTJP 4 (5) was isolated from the blast-infected wheat cv. Prodip (BARI Gom-24)’s spikelets of Jhenaidah in Bangladesh. For this work, a single spore culture was preserved at 4 °C on dried filter paper [26]. The isolate was re-cultured for 7 days on Potato Dextrose Agar (PDA) medium at 25 °C. Ten-day-old PDA-grown fungal cultures were cleaned in an aseptic condition in a laminar flow hood using 500 mL deionized water to exclude aerial mycelia and then maintained at room temperature (25–30 °C) for 2–3 days to stimulate profuse conidia formation [26,53]. After applying 15 mL of water to each plate, a glass slide was used to scrape out the conidia. The conidial and hyphal solution was filtered using double layered cheese cloth and set to 1 × 10^5^ conidia/mL in concentrations. Using a compound microscope, conidial germination was observed and counted. For the bioassay on leaves, wheat blast sensitive variety Prodip (BARI Gom-24) five-leaf phase seedlings were utilized [54,55,56].

### 2.2. Chemicals

**Figure 1:**
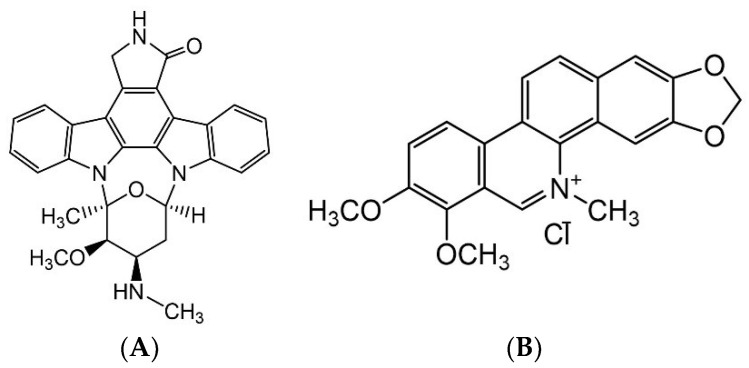
Chemical structures of (**A**) staurosporine and (**B**) chelerythrine chloride.

Staurosporine and chelerythrine chloride (Figure 1) were purchased from Sigma-Aldrich Co., St. Louis, MO, USA. The fungicide Nativo^®^75WG (1/2% mixture of trifloxystrobin and tebuconazole) was bought from Bayer Crop Science Ltd. in Dhaka, Bangladesh. Small volumes of DMSO (dimethyl sulfoxide) were utilized to prepare stock solutions of test compounds, which were subsequently diluted using water. The final solution contained no more than 1% (*v*/*v*) DMSO, which had no effect on MoT mycelial development or sporulation [55].

### 2.3. Mycelial Growth Suppression and Morphological Impacts on Mycelium

The mycelium growth inhibition of MoT isolate BTJP 4(5) by these PKC inhibitors and the commercial fungicide Nativo^®^75WG was determined using a modified disc diffusion method described by Chakraborty et al. [55]. Briefly, required amounts of alkaloids and the Nativo^®^75WG were dissolved in ethyl acetate and water to prepare a series of concentrations that ranges from 0.05 to 20 µg/disc. The test compound solutions were absorbed by nine-millimeter diameter filter-paper discs (Sigma-Aldrich Co., St. Louis, MO, USA). The treated discs were positioned 2 cm away from the one side of 9 cm diameter Petri dishes with 20 mL of PDA. The filter paper disc with the test compounds were put on the opposite side of the 5 mm diameter mycelial plugs of actively developing 7-day-old MoT cultures on PDA. As a positive control, Petri dishes inoculated with fungal hyphal plugs against Nativo^®^75WG were utilized. Filter paper discs were coated with ethyl acetate, and then evaporated in room temperature as a negative control. Amid 10 days of culture, a suppression of fungal hyphal growth was observed. The plates representing control treatment were kept at 25 °C until fungal culture covered the whole agar surface. Each concentration included five replications, and the test was conducted five times. Radial growth of the fungal culture was estimated in centimeters using a ruler and two perpendicular lines made on the lower edge of each plate. Inhibition zone formed by tested metabolites and the corresponding hyphal growth were measured and recorded. From mean data, the radial growth inhibition percentage (RGIP) (± standard error) [57] was determined as:

$$\begin{array}{l} \mathrm{RGIP}\;\%= \end{array}\frac{\begin{array}{l} \mathrm{Radial}\;\mathrm{growth}\;\mathrm{in}\;\mathrm{control}\;\mathrm{plate}\;-\;\mathrm{Radial}\;\mathrm{growth}\;\mathrm{in}\;\mathrm{treated}\;\mathrm{plate} \end{array}}{\mathrm{Radial}\;\mathrm{growth}\;\mathrm{of}\;\mathrm{control}}\;\begin{array}{l} \times \;100\% \end{array}$$

A digital camera of Canon DOS 700D was used to capture the disc diffusion test. A Zeiss Primo Star microscope was employed to examine the mycelial morphology at the sharp end of the cultures confronting the treated and the control discs at 40× and 100× magnification. A Zeiss Axiocam ERc 5s was utilized to acquire photos of the mycelium via the microscope.

### 2.4. Conidia Formation (Conidiogenesis) Suppression

Each PKC inhibitor’s stock solutions were prepared in 10 μL of DMSO and afterward diluted using distilled water to achieve concentrations of 50, 100, 200, and 300 μg/mL. The final solution contained no more than 1% (*v*/*v*) DMSO, which had no effect on MoT hyphal development or sporulation. The required quantity of material was mixed with distilled water to prepare a 5 mL solution of Nativo^®^75WG at concentrations of 50, 100, and 200, and 300 μg/mL serves as a positive control. We have established a conidiogenesis inhibition assay of MoT in our laboratory, which was followed by [55,56]. Shortly, a 10-day-old MoT Petri dish culture’s mycelium was washed to deplete nutrients and stimulate conidiogenesis [53,58]. MoT hyphal agar blocks of 10 mm were treated with 50 µL of each metabolite and Nativo^®^75WG at the aforementioned concentrations before being placed in Nunc multi-well plates. As a negative control, the same quantity of sterile water was added to the mycelial agar block of MoT with 1% DMSO. MoT hyphal agar blocks that had been treated were kept at 28 °C and >90% RH for 14 h of light and 10 h of darkness. With a Zeiss Primo Star microscope, conidiogenesis was studied at 40× magnification and after 24 h, photos were taken with a Zeiss Axiocam ERc 5s. With at least five replications per each treatment, the test was replicated five times.

### 2.5. Conidia Germination Inhibition and Morphological Abnormalities in Germinated Conidia

Stock solutions of every PKC inhibitor were made by dissolving 0.1 µg of compound in 10 µL of DMSO, then diluting with distilled water to make the concentration to 0.1 µg/mL. A 0.1 µg/mL solution of Nativo^®^75WG was made using distilled water, which serves as a positive control. We established a protocol for MoT conidial germination studies, which was followed by [55,56]. A 100 μL solution of 0.1 μg/mL was immediately mixed to 100 μL, containing 1 × 10^5^ MoT conidia/mL, to make a 200 μL of final solution in a hole of a 96-multi-well plate having test compounds of 0.05 μg/mL. The suspension was instantly mixed with a rod of glass before being incubated for 6 h, 12 h, and 24 h in a humid chamber at 25 °C. As a control, sterile water containing 1% DMSO was used. From each of the five replications, a sum of 100 conidia was studied at 100× magnification using a Zeiss Primo Star microscope. The photos were taken with a Zeiss Axiocam ERc 5s, and the percent conidia germination, as well as morphological modifications of spore germ tubes and appressoria, was assessed. The study was conducted five times with at least five replications per treatment. From mean results, conidia germination percentage (±standard error) was estimated as: CG% = (C − T)/C × 100; where, %CG = conidia germination, C = conidia germination percentage in control, and T = conidia germination percentage in treated samples.

### 2.6. Progression of Wheat Blast on Separated Wheat Leaves

Small amount of DMSO was utilized to prepare staurosporine and chelerythrine chloride stock solutions. The natural compounds were then prepared in distilled water at 50, 100, 200, and 300 µg/mL, with the final DMSO concentration never exceeding 1%. The concentrations of Nativo^®^75WG were also 50, 100, 200, and 300 µg/mL. Sterilized water containing 1% DMSO was used as a negative control. This experiment was also conducted following the methodology described by Chakraborty et al. [55,56]. At first, leaves of wheat were separated from seedlings at the five-leaf stage and put inside plates having 3 layers of damp paper towels. Three 20 µL drops of the properly synthesized test metabolites at the aforementioned concentrations were put on three distinct places of each leaf and allowed to dry for 15 min. After that, each location was inoculated with 1 µL conidial solution having 1 × 10^5^ MoT conidia/mL, and the dishes were incubated at 28 °C with 100% Rh in the dark for the first 30 h and then under continuous light for next two days. The test was carried out five times repeatedly with five different samples each time. The length of blast lesions caused by MoT was assessed from three leaves per study for each treatment and compound concentration.

### 2.7. Field Evaluation of Staurosporine and Chelerythrine Chloride for Wheat Blast Control

#### 2.7.1. Land Preparation, Seed Sowing, and Plot Maintenance

The experiment was carried out in a constrained area of the Bangabandhu Sheikh Mujibur Rahman Agricultural University (BSMRAU) research field in Gazipur, Bangladesh. The trial location was at 24.09 north latitude, 90.26 east longitude, and 8.4 m above mean sea level. The land was thoroughly ploughed and cleared of weeds and stubbles. During land preparation, adequate amounts of well-decomposed cowdung were applied. Urea, triple superphosphate, muriate of potash, and gypsum were applied as chemical fertilizers at ratios of 70-28-50-11 kg/ha [59]. All other fertilizers and two-thirds of the urea were administered as a baseline dosage 3–4 days before seed sowing at the final land preparation. The remaining one-third of the urea was applied at the first irrigation, 20 days after sowing (DAS). In the first week of December, wheat seeds of variety BARI Gom-26 were sown. Before sowing, the seeds were treated with Vitavex 200 (3 g/kg seed). The plots were all labeled properly. Irrigation and other intercultural activities were carried out as required. The experiment was carried out using a randomized complete block design (RCBD).

#### 2.7.2. Infection Assay in the Reproductive Phase of Wheat

Thoroughly prepared 5 µg/mL concentrations of the test compounds were sprayed on each plot and allowed to dry overnight, while sterilized water with 1% DMSO served as a negative control. Spore suspension was administered to wheat fields shortly after the flowering stage. The positive control was fungicide Nativo^®^75WG, whereas the negative control was deionized distilled water. Plots were coated with polyethylene sheets before inoculation to maintain a humid environment conducive to spore germination.

#### 2.7.3. Data Collection, Disease Intensity, and Severity Evaluation

During the reproductive phase, data on total tiller, productive tiller, and diseased tiller per hill, full length and affected portion of spike, seeds per spike, 1000-grain weight, and grain yield per hill were recorded. During the vegetative phase, data on total seedlings, diseased seedlings per pot, full length, and affected portion of the leaves were collected. The intensity of the disease (DI) was determined by employing the formula:

$$\mathrm{DI}=\frac{\begin{array}{l} \mathrm{Total}\;\mathrm{no}.\;\mathrm{of}\;\mathrm{infected}\;\mathrm{plants} \end{array}}{\mathrm{Total}\;\mathrm{no}.\;\mathrm{of}\;\mathrm{plant}\;\mathrm{observed}}\;\begin{array}{l} \times \;100\% \end{array}$$

Likewise, the severity of blast disease was assessed using a 5-point scale, with % infection referring to the length of the spike affected by blast. The scales were 0 for no lesions, 1 for 1–25% infection, 2 for 26–50% infection, 3 for 51–75% infection, and 4 for 76–100% length of infected leaves. The following formula was used to calculate the severity of the blast:

$$\mathrm{DS}=\frac{n\;\times \;v}{N\;\times \;V}\begin{array}{l} \times \;100\% \end{array}$$

where, DS = disease severity

n = number of leaves infected by blast
v = value score of each category attack
N = number of leaves observed
V = value of highest score

### 2.8. Experimental Design and Statistical Analysis

The fungicidal activity of the pure compounds was determined in the laboratory and in the field using a fully randomized design (CRD) and a randomized complete block design (RCBD), respectively. All statistical analyses were performed using IBM SPSS Statistics 25 and the Microsoft Office Excel 2015 application package. Tukey’s HSD (Honestly Significance Difference) test was used to compare the means of the treatments. Each treatment was repeated five times, and the mean value ± standard error was utilized in the tables and figures.

## 3. Results

### 3.1. Hyphal Growth Suppression and Morphological Impacts on Mycelium

**Figure 2:**
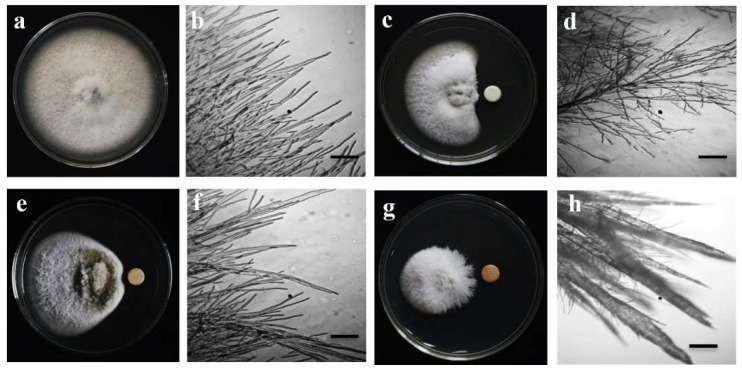
Photographs and micrographs of in vitro antifungal activity of staurosporine, chelerythrine chloride and a commercial fungicide, Nativo^®^75WG against wheat blast fungus, *Magnaporthe oryzae Triticum* (MoT) at 20 µg/disk. (**a**) Mycelial growth of MoT on PDA (control). (**b**) Normal tubular hyphal growth in a control. (**c**) Inhibition of MoT mycelial growth by chelerythrine chloride. (**d**) Disrupted tubular growth of MoT hyphae in the presence of chelerythrine chloride. (**e**) Inhibition of MoT mycelial growth by staurosporine. (**f**) Disrupted tubular growth and excessive branching in MoT hyphae in the presence of staurosporine. (**g**) Inhibition of MoT mycelial growth by Nativo^®^75WG. (**h**) Abnormal growth and necrosis in MoT hyphae in the presence of Nativo^®^75WG. Bar = 50 μm.

**Figure 3:**
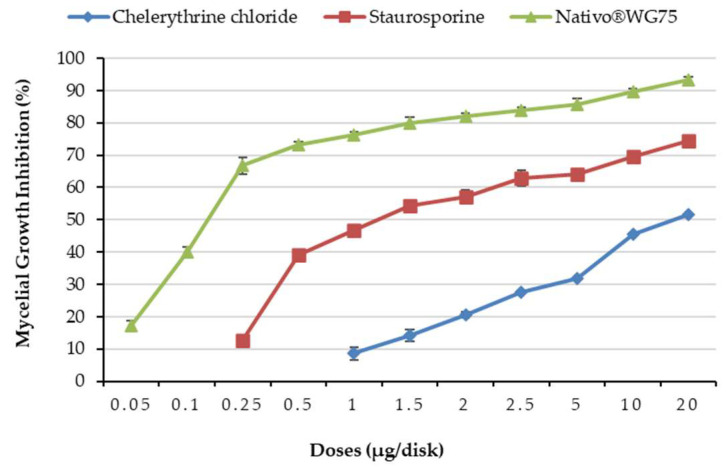
Inhibitory effects of chelerythrine chloride, staurosporine and the commercial fungicide, Nativo^®^75WG on mycelial growth of wheat blast fungus *Magnaporthe oryzae Triticum* (MoT) in potato dextrose agar media. The data are the mean ± standard errors of five replications for each concentration of the compound tested at 5% level based on the Tukey HSD (Honest Significance Difference) post-hoc statistic.

Both staurosporine and chelerythrine chloride (Figure 1) significantly inhibited MoT mycelium development on PDA (Figure 2). Staurosporine inhibited MoT mycelium development more efficiently than the other compound. When both staurosporine and chelerythrine chloride were administered at 20 µg/disk, hyphal growth suppression was 74.3 ± 1.6% and 51.6 ± 0.8%, respectively (Figure 3). Both staurosporine and chelerythrine chloride demonstrated lower inhibitory capacity than the commercial fungicide Nativo^®^75WG (93.3 ± 0.9% at 20 µg/disk).

Mycelial growth of MoT was inhibited by both PKC inhibitors in a dose-dependent manner. The suppressible effects of these alkaloids rose as concentrations increased from 0.05 to 20 μg/disk, attaining 74.3% for the staurosporine (Figure 3). Staurosporine inhibition was a bit lower than Nativo^®^75WG but greater than chelerythrine chloride suppression. At concentrations less than 0.1μg, both the alkaloids were inactive against MoT. Staurosporine impeded MoT hyphal growth extensively at 20 μg/disk (74.3 ± 1.6%), 10 μg/disk (69.5 ± 1.1%), and 5 μg/disk (64.1 ± 1.2%), indicating a positive interaction between inhibition and accelerated concentrations. At 20, 10, and 5 μg/disk, chelerythrine chloride suppressed 51.6 ± 0.8%, 45.5 ± 1.8%, and 31.7 ± 1.9% hyphal growth of MoT.

Staurosporine and chelerythrine chloride had minimal inhibitory concentrations of 0.25 μg/disk and 1 μg/disk, respectively. Staurosporine and chelerythrine chloride inhibited hyphal development by 12.6 ± 1.9% and 8.62 ± 1.6% at 0.25 and 1 μg/disk, respectively. Furthermore, 0.05 μg/disk was the minimum inhibitory concentration of Nativo^®^75WG.

Untreated MoT mycelium displayed polar, tubular development with smooth, branching, hyaline, septate, plump, and unbroken mycelium under microscopic investigations (Figure 2a). Staurosporine and chelerythrine chloride-treated hyphae developed irregularly and had a greater frequency of branching per unit of mycelium length (Figure 2b,c). The fungicide Nativo^®^75WG inhibited hyphal development in a similar way. When the mycelia were near to the filter disc of Nativo^®^75WG, a similar anomaly of MoT hyphae occurred (Figure 2d). However, the two PKC inhibitor alkaloids caused slightly distinct morphological abnormalities in MoT compared to the Nativo^®^75WG, indicating a potentially different mode of action.

### 3.2. Conidia Formation (Conidiogenesis) Suppression

**Figure 4:**
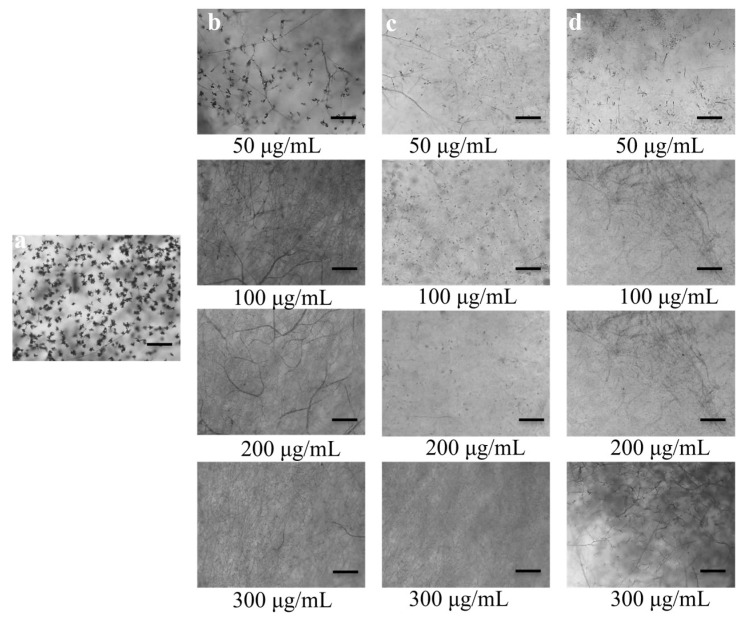
Effects of staurosporine, chelerythrine chloride, and the fungicide Nativo^®^75WG on inhibition of conidiogenesis of *Magnaporthe oryzae Triticum* in 96-multi-well plates at 50 μg/mL, 100 μg/mL, 200 μg/mL, and 300 μg/mL. (**a**) Profuse production of conidia on conidiophores (conidiogenesis) in untreated control. Suppressed conidiogenesis by varying doses of staurosporine (**b**), chelerythrine chloride (**c**), and Nativo^®^75WG (**d**). Bar = 50 μm.

When compared to the control, PKC inhibitors and fungicide significantly reduced conidial production by MoT at concentrations of 50, 100, 200, and 300 µg/mL, and suppression increased as concentrations climbed from 50 to 300 µg/mL (Figure 4). In all three treatments, less or no conidia was produced at 300 µg/mL. For all three treatments at 300 µg/mL, microscopic investigation exhibited damaged hyphal tips and a total absence of conidiophores.

### 3.3. Conidia Germination Inhibition and Morphological Abnormalities in Germinated Conidia

In the multi-well plates, staurosporine, chelerythrine chloride, and Nativo^®^75WG at 0.05 μg/mL were employed to investigate the inhibition of MoT conidial germination. The rate of germinated conidia was measured after 6, 12, and 24 h of incubation (Table 1). When compared to control, all three treatments dramatically inhibited conidia germination after 6 h. In water, 100% of conidia were germinated, whereas in Nativo^®^75WG treated plates, it was 51.6 ± 0.8%. At 0.05 μg/mL, the germination rates of MoT conidia with staurosporine and chelerythrine chloride were 27.1 ± 0.8% and 67.5 ± 0.7%, respectively.

**Figure 5:**
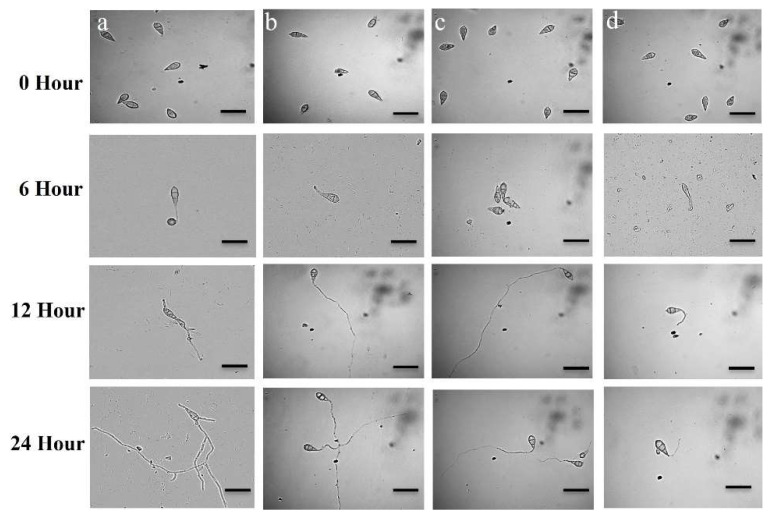
Time-dependent observation of germination of conidia of *Magnaporthe oryzae Triticum* and their subsequent morphological changes in the control plate (**a**), presence of staurosporine (**b**), chelerythrine chloride (**c**), and the commercial fungicide, Nativo^®^75WG (**d**). Dose of chelerythrine chloride, staurosporine, and Nativo^®^75WG was 0.05 μg/mL. Bar = 10 μm.

At all the incubation durations (6 h, 12 h, and 24 h), 100% of conidia was germinated in water, with typical germ tube formation and hyphal development (Table 1, Figure 5a) in the dark at 25 °C. At 0.05 µg/mL, the two PKC inhibitors showed negative impacts on conidial germination and post-germination developmental activities, inducing aberrant transitions from one phase to the other.

During the 6 h of incubation under the influence of staurosporine, 27.1 ± 0.8% of conidia germinated with a small germ tube. After 12 h of the same treatment, 4.7 ± 0.8% germ tubes were normal, whereas 18.1 ± 0.6% were abnormally long hyphae-like germ tubes. After 24 h, there were no appressoria and hyphal development took place (Table 1, Figure 5b).

In the case of chelerythrine chloride, 15.2 ± 0.7% of the conidia were lysed after 6 h, and 52.3 ± 0.8% germination occurred with a short germ tube after 6 h (Table 1, Figure 5c). After 12 h, 13.3 ± 0.8% normal and 32.5 ± 0.6% abnormally long hyphae-like germ tube were formed (Table 1, Figure 5c). There was no appressoria formation and hyphal growth occurred after 24 h (Table 1, Figure 5c).

After 6 h and 12 h with the influence of Nativo^®^75WG, 51.6 ± 0.8% of conidia germinated with typical germ tubes, no appressoria formed. After 24 h, the fungicide Nativo^®^75WG also suppressed sporulation (Table 1, Figure 5d). It is worth noting the fact that the alkaloids induced abnormally long hyphae-like germ tubes and conidia lysis, whilst the fungicide had no such effects.

### 3.4. Progression of Wheat Blast on Separated Wheat Leaves

**Figure 6:**
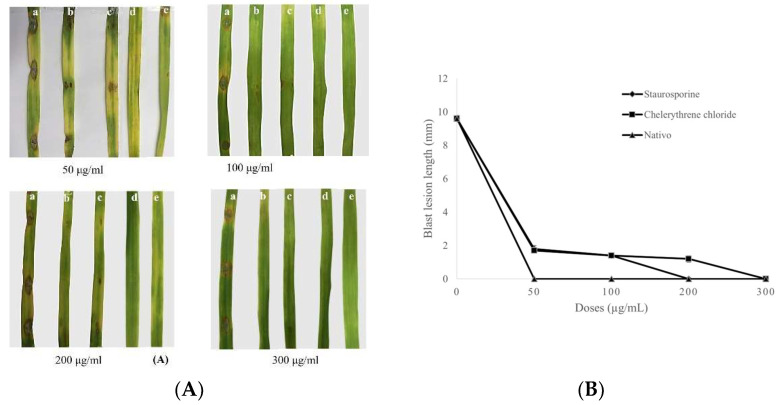
Suppression of wheat blast disease by staurosporine, chelerythrine chloride at 50 μg/mL, 100 μg/mL, 200 μg/mL, and 300 μg/mL on representative detached wheat leaves inoculated with *Magnaporthe oryzae Triticum*. (**A**) Blast lesions on treated and untreated wheat leaves. (a) MoT inoculation after treatment of leaf with water (control). (b) MoT inoculation after treatment of leaf with staurosporine. (c) MoT inoculation after treatment of leaf with chelerythrine chloride. (d) MoT inoculation after treatment of leaf with Nativo^®^75WG. (e) Non-inoculated after treatment with water. The experiment was repeated 5 times and only the representative images are shown. (**B**) Blast lesion lengths on detached wheat leaves treated with staurosporine, chelerythrine chloride, and Nativo^®^75WG fungicide compared with water treatment (control). The data are the averages ± standard errors of at least five replicates for each dose of the tested compounds at *p* ≤ 0.05. Bars represent ± standard error.

The two PKC inhibitors applied at 50, 100, 200, and 300 µg/mL significantly reduced symptoms of wheat blast observed in separated wheat leaves infected with MoT. The lesion length in wheat leaves treated with staurosporine averaged 1.8 ± 0.2 mm at 50 µg/mL, 1.4 ± 0.1 mm at 100 µg/mL, and 0 ± 0 mm at 200 and 300 µg/mL, respectively (Figure 6A,B). The blast lesion lengths with chelerythrine chloride were 1.7 ± 0.1 mm, 1.4 ± 0.1 mm, 1.2 ± 0.2 mm, and 0 ± 0 mm at 50, 100, 200, and 300 µg/mL, respectively (Figure 6A,B).

Leaves of wheat treated with Nativo^®^75WG at 50, 100, 200, and 300 µg/mL showed no signs of blast (Figure 6A,B). Neither of the compounds developed signs at 300 µg/mL. Water-treated leaves exhibited normal blast lesions, having length averaging 9.6 ± 0.2 mm (Figure 6A,B) as a negative control. The fungicide inhibited lesion formation more than both the alkaloids at all the applied concentrations.

### 3.5. Wheat Blast Disease Suppression by PKC Inhibitors at the Heading Stage of Wheat in the Field

**Figure 7:**
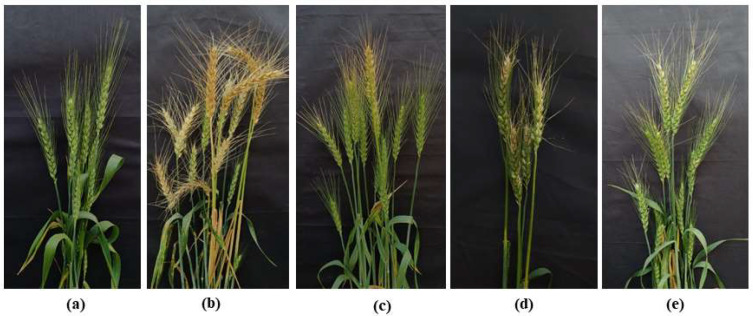
Suppression of wheat blast symptoms with staurosporine and chelerythrine chloride at 5 μg/mL. Herein, (**a**) Non-inoculated, non-treated spike, (**b**) Water control + MoT inoculation, (**c**) Staurosporine + MoT inoculation, (**d**) chelerythrine chloride + MoT inoculation, (**e**) Nativo^®^75WG + MoT inoculation.

Wheat blast is a disease that mostly affects the heads of wheat. A field experiment was carried out with a commercial fungicide, Nativo^®^75WG at 5 µg/mL, to determine if these alkaloids inhibit blast disease in artificially infected wheat spikes. In the field, staurosporine and chelerythrine chloride significantly decreased the wheat blast incidence (36.33% and 51.33%, respectively) (Figure 7c,d, Table 2), whereas the blast disease incidence was 91.33% in the untreated control plot (Figure 7b, Table 2).

Furthermore, wheat plants treated with these natural products showed blast severity of 28.67% and 40.33%, respectively, compared with 83.3% of untreated control. When comparing with the untreated control (56.4 ± 3.67 gm), staurosporine (120.6 ± 2.62 gm), chelerythrine chloride (117.2 ± 3.56 gm), and Nativo^®^75WG (1316.63 gm) exhibited considerably higher grain yields. The grain yields in the staurosporine, chelerythrine chloride, and Nativo^®^75WG treatments were similar to the healthy control (136.6 ± 3.9 gm) (Table 2).

One thousand grain weights were also measured for each treatment and weights of 45.28, 36.10, 42.06, and 50.90 gm were obtained for Nativo^®^75WG, chelerythrine chloride, staurosporine, and the negative control plot, respectively. These grain yields were much higher than the untreated control plot’s yield (34.12 gm) (Table 2).

## 4. Discussion

In this study, we observed that the alkaloid antibiotics staurosporine and chelerythrine chloride have potent antifungal activity against *M. oryzae Triticum* (MoT), a destructive pathogen of wheat blast. The results of in vitro bioassays indicated that the chemicals significantly decreased hyphal development, conidia formation, germination and developmental changes of germinated conidia, and controlled wheat blast in vivo. Consequently, our data suggested that these alkaloids can decrease wheat blast disease in vivo by inhibiting hyphal proliferation and conidial germination. Many natural compounds have been documented to inhibit mycelial development, conidia formation, and conidial germination of different fungi, including rice blast and wheat blast fungus on an in vitro bioassay [55,56,58,60,61,62,63,64]. Moreover, alkaloid antibiotics have a wide spectrum of antimicrobial [58,65,66], anti-inflammatory [67], antitumor [68], anti-angiogenesis [69], and anti-acetylcholinesterase properties [70]. Furthermore, these alkaloids are potent inhibitors of Ca^2+^/phospholipid-dependent protein kinase or protein kinase C (PKC), which is a major modulatory enzyme in signaling pathways governing different cellular responses of eukaryotes [2,12,20]. Despite the PKC inhibitor alkaloids’ exceptional biological features, only a few studies have concentrated on their use as plant protection agents to far. It is, however, the first report of natural bioactive PKC inhibitor alkaloids derived from *Streptomyces* spp. controlling MoT.

The hyperbranching phenomenon of MoT hyphae by these alkaloids is one of the most remarkable findings in our work (Figure 2b), where we used doses varying from 0.005 to 2 µg/disk. With higher concentrations, branch frequency increased proporationately. Phloroglucinols and xanthobaccin A have been found to cause hyperbranching in fungal mycelium [71,72,73,74]. Magae and Magae [75] reported that staurosporine restricted mycelial growth of *Pleurotus ostreatus* in a different way than that of MoT. It induced stubby and balbous hyphae as well as swelling of hyphal tips and subapical regions. On the other hand, chelerythrine inhibited the mycelial growth of *Ustilaginoidea virens* by inducing thin, twisted, atrophied, narrow, and locally fractured mycelium [76]. According to Yang et al. [77], chelerythrine derivatives extracted from *M. microcarpa* greatly suppressed the hyphal development of *Curvularia lunata*, *Fusarium solani*, *Valsa mali*, *Fusarium oxysporum f. sp. vasinfectum*, *Alternaria alternate*, *Pyricularia oryzae*, and *Fusarium oxysporum*. Thus far, this is the foremost evidence-based report of two PKC inhibitor alkaloids exhibiting hyperbranching-like predatory behaviour against a damaging fungal pathogen. More research is requisite to detecting the method of action of these alkaloids against the dangerous wheat pathogen MoT.

Conidia are fungal spores that are generated asexually on a conidiophore, and the method of forming conidia is termed conidiogenesis [62]. Most of the plant pathogenic fungi attack plant conidia or spores [78]. The probability of invasive fungal infections is lessened when conidiogenesis and conidia germination are inhibited. Compounds that impede these processes have the potential to be used as plant protection solutions in the future. Another intriguing discovery of this work is that these PKC inhibitors significantly reduced conidiogenesis (Figure 5), germination and triggered developmental changeover of MoT conidia (Table 2, Figure 6). Conidia lysis and unusually long hypha-like conidial germ tubes were among the other unique and interconnected phenomena discovered in this work (Figure 5b,c). A similar occurrence was discovered by oligomycins derived from *Streptomyces*, which trigger lysis of conidia and develop hyphae-like germ tubes of phytopathogen MoT [56]. Islam and his colleagues [79] discovered that staurosporine and chelerythrine chloride suppress zoosporogenesis, hamper motility, and trigger lysis of *Plasmopara viticola* zoospores. Lecithin caused aberrant branching germ tube tips and hindered the formation of appressoria in rice blast fungus, according to Homma and his co-workers [80]. Methods of action and modes of suppressing conidiogenesis, conidia, and appressoria formation in MoT conidia by alkaloids have not previously been documented.

The PKC inhibitors staurosporine and chelerythrine chloride have antimicrobial activities against several bacteria, fungi, and peronosporomycetal plant pathogens, including *Xanthomonas oryzae*, *Staphylococcus aureus*, *Pleurotus ostreatus*, *Phytophthora apsica*, *Valsa mali*, *Fusarium solani*, *Alternaria alternate*, *Pyricularia oryzae*, *Candida* sp., *Saccharomyces* sp., *Aspergillus* sp. *Fusarium oxysporum*, *Plasmopara viticola*, *Aphanomyces cochlioides*, etc. [7,75,77,79,81,82,83]. Staurosporine was discovered as a novel antifungal alkaloid synthesized by *Streptomyces* sp. AM-2282 [7]. Staurosporine is a selective inhibitor which binds tightly to the ATP domain of nearly all active kinases [84,85,86]. It is a powerful inhibitor of PKC as well as other tyrosine, serine, and threonine protein kinases [87,88,89]. Staurosporine has also been identified as a potential promoter of plant resistance as well as a modulator of genes involved in causing programmed cell death or the production of defence factors within the host [82]. Staurosporine has a severe cytotoxic impact on mammalian cell growth (IC_50_ values: 90 nM for MCF-7 cells, <3 nM for HeLa S3 cells) [90]. Furthermore, chelerythrine is a natural benzophenanthridine alkaloid obtained from herbal plants. Chelerythrine, unlike staurosporine, is at least 100 times more specific for PKC than other kinases (i.e., PKA, PKG) [17,18]. Chelerythrine’s selectivity for PKC has led to its application in studies of PKC activities in cells [91]. Chelerythrine is mostly desirable for its kinase selectivity, simplicity of application in situ, capacity to induce ceramide formation, and ATP-independent inhibition of PKC [17,18,92]. The inhibitory activities of chelerythrine on DNA synthesis, proteinase production, and membrane permeability are also linked to its antimicrobial modes of action [76,83]. Several studies have found that chelerythrine stimulates intracellular ROS formation, which leads to apoptosis [76,93,94,95]. It has also been reported as a BclXL-Bak BH3 peptide binding inhibitor. It triggered apoptosis in BclXL-overexpressing cells that have been entirely resistant to staurosporine by releasing Cytochrome C from the isolated mitochondria [96]. Chelerythrine chloride is cytotoxic to tumor cell lines of human [18,93]. Islam et al. [79] confirmed that inhibitory activity of staurosporine and chelerythrine chloride is linked with PKC inhibition in the cells. The outcomes of this work do not specify the details mechanism of action, but they do suggest that inhibition of PKC activities in cells could suppress the hyphal development and impede conidia germination as these cellular processes require supply of energy from mitochondria and intracellular Ca^2+^ ions [97]. Elucidating the activities of PKC in suppressing hyphal development, conidia formation, conidial germination, and appressoria production might help us comprehend the biology and the pathogenicity of filamentous plant pathogens. Therefore, these PKC inhibitors from natural source might be a potential pioneer compound for producing new, effective agrochemicals to combat this notorious plant pathogen.

The application of these PKC inhibitors effectively inhibited blast disease of wheat in in vivo experiments, which was also a noteworthy finding in this investigation (Figure 6 and Figure 7). In this work, wheat leaves that had previously been treated with those alkaloids exhibited smaller lesions in length than the untreated controls (Figure 6). Most of those lesions were tiny, brown patches with pinhead-sized spots (scale 1) to tiny, roundish to moderately extended, grey dots measuring 1–2 mm in diameter (scale 3). According to the IRRI Standard Evaluation System’s 9-scale blast disease evaluation system [98], the untreated control leaves displayed normal blast lesions spreading 26–50% of the leaf surface (scale 7). With the influence of Nativo^®^75WG, nevertheless, no apparent lesions of blast were observed. We found comparable findings at the wheat heading stage. On artificially infected wheat spikes, staurosporine and chelerythrine chloride substantially suppressed the development of blast disease (Figure 7). Herein, Nativo^®^75WG is a commercially available systemic broad-spectrum fungicide that was employed as the positive control. These alkaloids have antifungal action similar to this fungicide when it relates to inhibiting the MoT fungus. Tebuconazole and the trifloxystrobin are the active ingredients in Nativo^®^75WG. Tebuconazole is a dimethylase inhibitor that is utilized as a systemic triazole fungicide (DMI). This impedes conidia germination and fungal growth by interfering with sterol production in fungal cell walls [99]. Trifloxystrobin is a fungicide of strobilurin that impedes the development of plant pathogenic fungi by interrupting metabolism and blocking electron transfer in mitochondria [100]. Although a comparable disease inhibition response has been noticed, the principles of actions of disease inhibition by these PKC inhibitors are probably distinct from those of Nativo^®^75WG. The fundamental mechanism by which these alkaloids inhibit wheat blast requires more research. Moreover, when recognizing these PKC inhibitors as efficient fungicides for wheat blast, a field study of their effectiveness in inhibiting wheat head infection is required.

Fungicidal resistance in phytopathogens is constantly evolving, necessitating the research and development of novel fungicides with an alternative mode of action. Natural product-based plant protectants have received much interest from scientists in recent decades because they are believed to be less hazardous to mammals and the environment [48,101]. These PKC inhibitors exhibit greater bioactivity than the commercialized fungicide Nativo^®^75WG, and the compound’s inhibitory efficacy against MoT should make it a candidate agrochemical with a novel method of action toward this plant pathogenic fungus.

## 5. Conclusions

Our findings revealed that two PK inhibitors, staurosporine and chelerythrine chloride, inhibited hyphal growth and asexual life phases of MoT fungus in vitro, as well as wheat blast disease in both leaves and spikes. To confirm if these metabolites are efficient fungicides towards wheat blast disease, a large-scale field evaluation of these alkaloids is required. More research is also required to elucidate the underlying molecular mechanisms and structure–activity relationship between these PK inhibitors and the wheat killer fungus *M. oryzae Triticum*.

## Figures and Tables

**Table 1:** Effects of staurosporine and chelerythrine chloride on Germination of Conidia and Their Subsequent Developmental Transitions of *Magnaporthe oryzae Triticum* (MoT) at the Dose of 0.05 µg/mL In Vitro.

Compound	Time (h)	Effects of Natural Compounds on Developmental Transitions of Conidia of Wheat Blast Fungus *M. oryzae Triticum* (MoT)	
		Germinated Conidia (% ± SE ^a^)	Major Morphological Change/Developmental Transitions in the Treated Conidia
Water	0	0 ± 0 e	No germination
	6	100 ± 0 a	Germinated with normal germ tube and normal appressoria developed
	12	100 ± 0 a	Mycelial growth took place
	24	100 ± 0 a	Huge mycelial growth took place
Staurosporine	0	0 ± 0 e	No germination
	6	27.1 ± 0.8 d	Germinated with a short germ tube
	12	22.8 ± 0.6 d	4.7 ± 0.8% Normal and 18.1 ± 0.6% abnormally long hyphae-like germ tube formed
	24	0 ± 0 b	No appressoria formation and mycelial growth took place
Chelerythrine Chloride	0	0 ± 0 e	No germination
	6	67.5 ± 0.7 b	Germinated with 52.3 ± 0.8% short germ tube and 15.2± 0.7% conidia lysis occurred
	12	45.8 ± 0.5 c	13.3 ± 0.8% Normal and 32.5± 0.6% abnormally long hyphae-like germ tube formed
	24	0 ± 0 b	No appressoria formation and mycelial growth took place
Nativo^®^75WG	0	0 ± 0 e	No germination
	6	51.6 ± 0.8 c	Germinated with a short germ tube
	12	51.6 ± 0.8 b	Normal germ tube formed
	24	0 ± 0 b	No appressoria formation or mycelial growth took place

^a^ Data presented here are mean value ± SE of three replications in each compound. Means within a column followed by the same letter(s) are not significantly different as assessed by Tukey’s HSD (honest significance difference) post-hoc (*p* ≤ 0.05).

**Table 2:** Effect of staurosporine and chelerythrine chloride on Yield and Yield Components of the Wheat Variety BARI Gom-26 under Field Condition after Artificial Inoculation with Wheat Blast Fungus.

Treatment	Yield/Plot (gm) *	1000-Grain WEIGHT (gm) *	Disease Incidence (%) *	Disease Severity (%) *
Healthy control	136.60 ± 0.3.98 a	50.90 ± 0.81 a	0.00 ± 0.00 d	0.00 ± 0.00 e
Untreated control	56.40 ± 3.67 c	34.12 ± 1.74 cd	91.33 ± 2.03 a	83.00 ± 3.06 a
Staurosporine	120.60 ± 2.62 b	42.06 ± 1.71 bc	36.33 ± 1.76 c	28.67 ± 1.45 c
Chelerythrine chloride	117.20 ± 3.56 b	36.10 ± 2.48 dcd	51.33 ± 1.74 b	40.33 ± 1.76 b
Nativo^®^75WG	131.00 ± 6.63 ab	45.28 ± 2.35 ab	30.00 ± 2.35 c	14.33 ± 2.40 d

* Any two means having a common letter are not significantly different at the 5% level of significance.

## Data Availability

All data are included in the manuscript.
